# Isolated aglossia in a six year old child presenting with impaired speech: a case report

**DOI:** 10.4076/1757-1626-2-7926

**Published:** 2009-09-17

**Authors:** Altaf Rasool, Mohammad Inam Zaroo, Adil Hafeez Wani, Mohammad Ashraf Darzi, Shiekh Adil Bashir, Akram Hussain Bijli, Shafaq Rashid

**Affiliations:** Department of Plastic and Reconstructive Surgery, Sher-i-Kashmir Institute of Medical SciencesSrinagar, KashmirIndia

## Abstract

**Introduction:**

The most mobile organ of the body, the tongue is associated with various congenital anomalies; most of which are in association with many other systemic abnormalities. Rarely do they occur in isolation. Isolated aglossia, that we presented, is one of the more rare presentations.

**Case presentation:**

Our patient is a 6-year-old male child of Asiatic origin from Kashmir (India), who was physically well built and mentally sound and presented with history of impaired speech. The patient had normal velopharangeal competence but absence of tongue which was replaced by a small mucus membrane projection near the floor of oropharangeal isthmus. The patient had no difficulty in feeding or taste sensation but he was unable to pronounce lingual consonants.

**Conclusion:**

Isolated aglossia is very rare condition explained on the basis of growth failure of lateral lingual swellings and tubercular impar. Such patients do not usually need reconstruction of tongue; as feeding, swallowing and taste sensations are usually intact and speech cannot be improved by reconstruction. However, malocclusion of teeth needs to be taken care of.

## Introduction

The tongue is the most mobile organ of the body, which helps in swallowing and speech. It has a special property of taste sensation and in addition helps in normal development of teeth and mandible. Various inherited, congenital, and developmental anomalies of tongue have been reported. Some are of minor clinical significance like ankyloglossia, fissured or scrotal tongue, and lingual thyroids. Some are of major severity and cause discomfort to person and affect their normal living pattern. Hypoglossia or microglossia is one of the more rare congenital anomalies manifested by the presence of a small or rudimentary tongue. Aglossia, the complete absence of tongue since birth, has also been reported in literature [1]. Most of these anamolies are in association with many other systemic abnormalities [2-7]. Rarely do they occur in isolation [8,9].

## Case presentation

**Figure 1.**
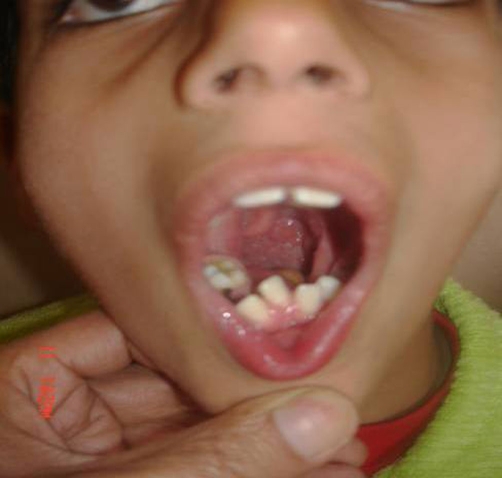
Photograph showing absent tongue malaligned teeth.

A 6-year-old male child of Asiatic origin from Kashmir (India) presented to our department with history of impaired speech. On detailed examination he was found to be physically well built and mentally sound. Local examination revealed normal velopharangeal competence but with absence of tongue which was replaced by a small mucus membrane projection near the floor of oropharangeal isthmus. Patient had no difficulty in feeding or taste sensation but he was unable to pronounce lingual consonants. He had hypoplastic mandible with class 2 malocclusion (Figure 1). There were no systemic or limb anamolies associated with it.

**Figure 2.**
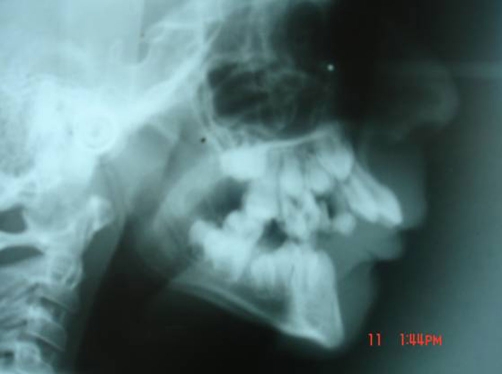
X-ray lateral face showing hypoplastic mandible, Malaligned teeth and marked overjet.

Radiology revealed hypoplasia of the mandible with malalignment of teeth (Figure 2).

## Discussion

Aglossia is a known entity but is usually associated with various craniofacial and limb abnormalities. Hall has given the following detailed classification for such anomalies (Table 1).

**Table 1.** Hall's classification (Oromandibular limb hypogenesis syndrome)

**Type-I**	
A	Hypoglossia
B	Aglossia
**Type-II**	
A	Hypoglossia - hypodactylia
B	Hypoglossia - hypomelia
C	Hypoglossia - hypodactylomelia
**Type-III**	
A	Glossopalatine ankylosis (Ankylossum Superius syndrome)
B	With hypoglossia
C	With hypoglossia - hypodactylia
D	With hypoglossia - hypomelia
E	With hypoglosia - hypodactylomelia
**Type-IV**	
A	Intraoral bands and fusion
B	With hypoglossia
C	With hypoglossia - hypodactylia
D	With hypoglossia - hypomelia
E	With hypoglossia - hypodactylomelia
**Type-V**	
A	The Hanhart syndrome
B	Charlie M-Syndrome
C	Pierre-Robin syndrome
D	Mobius syndrome
E	Amniotic band syndrome

As per this classification, our patient is catagorized as Type-1B. Various congenital malformations along with aglossia have been reported. Preis et al noticed anodontia, epidermoid of right eye, 6th and 7th nerve palsy, left hypodactyli and VSD along with aglossia [2]. Kantapura et al described a case where aglossia was associated with developmental delay in milestones, hypothyroidism, microcephaly, microsomia, collapse of mandibular arch, congenital absence of mandibular teeth along with persistence of buccopharangeal membrane [3]. A two day old neonate was detected by Mandai et al [4], during intubation for jejunal atresia, to have aglossia-adactalia along with cleft palate. Aglossia with severe abnormalities of structures derived from first branchial arch has also been reported [5].

Isolated aglossia is a rarity, reported only by few [8,9]. Our patient was a six-year-old male child who presented with slurring of speech and inability to pronounce the lingual consonants clearly. Normal feeding and swallowing in the child depicts the adaptability of oral structures to such function but not to speech. Normal taste sensations could be because of presence of taste buds within the floor of mouth and in the mucus membrane projection present at isthmus. This mucus membrane projection is actually a remnant of persistent embryonic oropharangeal membrane. Presence of maldevelopment of mandible and malocclusion of the teeth in our patient is because of absence of muscular pressure created by the normal tongue and is usually a part of long standing isolated aglossia.

## Conclusion

It is concluded that isolated aglossia is very rare condition explained on the basis of growth failure of lateral lingual swellings and tubercular impar, the actual etiology of which remains largely unknown [10]. Such patients do not usually need reconstruction of tongue; as feeding, swallowing and taste sensations are usually intact and speech cannot be improved by reconstruction. However, malocclusion of teeth needs to be taken care of.
